# Microphthalmia and cataract in rats with a novel point mutation in connexin 50 - L7Q

**Published:** 2008-05-07

**Authors:** František Liška, Blanka Chylíková, Jindřich Martínek, Vladimír Křen

**Affiliations:** 1 Institute of Biology and Medical Genetics of the 1st Faculty of Medicine and General Teaching Hospital, Charles University in Prague, Prague, Czech Republic; 2 Institute of Histology and Embryology, 1^st^ Faculty of Medicine, Charles University in Prague, Prague, Czech Republic; 3 Institute of Physiology, Academy of Sciences of the Czech Republic, Prague, Czech Republic

## Abstract

**Purpose:**

We isolated an autosomal semi-dominant cataract from our inbred SHR/OlaIpcv rat colony. Heterozygotes express pulverulent cataract with smaller eyes; homozygotes express marked microphthalmia with hypoplastic lens. We call this mutation *Dca* (for dominant cataract). In this study, we focus on the identification of the responsible gene.

**Methods:**

We performed linkage mapping using 93 F2(SHR-*Dca* x PD) hybrids and a panel of microsatellite markers. In a separate group of animals with a SHR genetic background, we examined the lenses histologically using Epon semi-thin sections and toluidine blue staining. We also assessed the weight of the eyes as an immediate measure for microphthalmia.

**Results:**

We mapped the *Dca* gene to chromosome 2, spanning 8.6 Mbp between markers D2Rat134 and D2Rat186. By sequencing the most plausible candidate gene, *Gja8* (coding for connexin 50), we found a T to A transversion at codon 7, leading to a substitution of glutamine for leucin (L7Q). L7Q lies within the NH_2_-terminal cytosolic domain, presumably involved in voltage gating. Histology revealed disturbances in cell to cell contacts in the lens.

**Conclusions:**

L7Q is a novel mutation in connexin 50 (*Gja8*), causing semi-dominant pulverulent cataracts. *Dca* rats can serve as a model for cataract development. A study on the properties of the mutant protein may offer an insight into the connexin channel function.

## Introduction

Congenital cataract is an important cause of vision impairment and blindness, accounting to about 1/10 of childhood blindness with incidence slightly above 2/10,000 live births. Half of congenital cataract cases are believed to be inherited; most often in autosomal dominant fashion [1].

**Figure 1:**
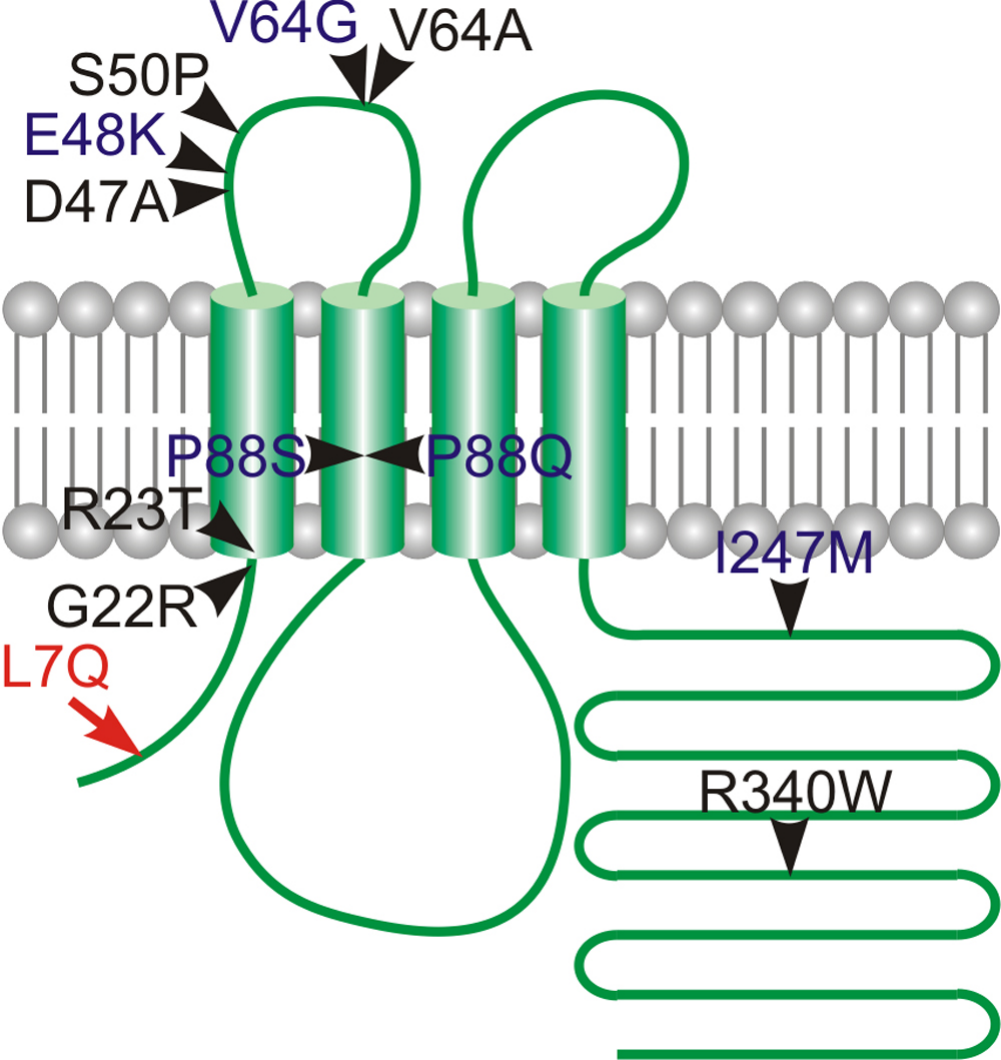
Connexin 50 mutations known to date. The point mutations in Cx50 causing the dominant form of microphthalmia and cataract in the rat, mouse, and human are shown: G22R (*Lop10* mouse) [15], R23T (human) [16], D47A (*No2* mouse) [17], E48K (human) [18], S50P (*L1* mouse) [19], V64A (*Aey5* mouse) [20], V64G (human) [21], P88S (human) [22], P88Q (human) [23], I247M (human) [24], and R340W (*Uca* rat) [25]. L7Q mutation affects the NH_2_-terminal cytosolic portion of Cx50 (arrow).

An increasing number of genes has been implicated in the genesis of cataract in humans as well as in model organisms (204 genotypes in Mouse Genome Informatics). These genes exhibit pronounced diversity with mutations ranging from the major structural constituents of the lens crystallins [2], intermediate beaded filaments phakinin and filensin [3], gap junction proteins like connexin 46 [4] and 50 (Figure 1 and [5-15]), and other membrane transport proteins like aquaporin Mip [16] to transcription factors like Pax6 [17], Maf [18], or Hsf4 [19], see also a review [20].

Three different connexins are expressed in the lens, α1 (Cx43), α3 (Cx46), and α8 (Cx50) [21]. Connexin 50 or α8 connexin (Cx50), coded by the *Gja8* gene (Gap junction membrane channel protein alpha-8), is expressed in the lens fiber cells as well as in the lens epithelial cells [22]. Cx50 fulfills an important role in the gap junctions in the eye. Null alleles (either generated by gene targeting [23,24] or a frameshift mutation [25]) result in recessive microphthalmia and pulverulent nuclear cataracts whereas several point mutations are associated with identical phenotype with a semi-dominant mode of inheritance (Figure 1 and [5-15]). Most of the identified point mutations are localized in the first extracellular loop, two are in the second transmembrane domain, two are in the COOH-terminal cytosolic domain, one is in the second transmembrane domain, and one is in the NH_2_-terminal domain (Figure 1).

Here, by using a classical positional cloning approach, we uncover a new mutation in the NH_2_-terminal cytosolic domain of Cx50, L7Q, which causes microphthalmia and cataract in a new mutant rat strain, SHR-*Dca* (for dominant cataract). This rat strain is derived from the spontaneously hypertensive rat (SHR) inbred strain; the cataract is inherited in autosomal-semi-dominant fashion. Interestingly, the NH_2_-terminal domain of Cx50 was reported to form part of the voltage sensor and therefore is thought to play a fundamental role in controlling the channel conductance [26].

## Methods

### Animals

In this study, the following rat inbred strains were employed: SHR/OlaIpcv, RGD (Rat Genome Database) ID: 631848; PD/Cub, RGD ID: 728161; the mutant strain SHR-*Dca*/Cub, BN/Cub, RGD ID: 737899; CHOC/Cub, RGD ID: 737958. All experiments were performed in agreement with the Animal Protection Law of the Czech Republic (311/1997), which is in compliance with the European Community Council recommendations for the use of laboratory animals 86/609/ECC. All experiments were approved by the Charles University Animal Care Committee.

### Inheritance assessment

To estimate inheritance of the cataract phenotype, we first crossed the cataract founder with the SHR/OlaIpcv control and then crossed their offspring that displayed the cataract phenotype. In the offspring of the second cross, we observed a distinct phenotype that can be described as microphthalmia. Next, we crossed the animals showing microphthalmia with the SHR/OlaIpcv control and then crossed their offspring displaying microphthalmia. The results (see Results section) allowed us to categorize the phenotype of the animals into wild-type, cataract, and microphthalmia and to assign them genotypes *+/+*, *+/Dca*, and *Dca/Dca*, respectively. We used the same visual categorization for linkage mapping.

### Linkage mapping

SHR-*Dca*/Cub was crossed with PD/Cub. F1 hybrids were intercrossed, and 93 F2 hybrids were obtained. At weaning, the eye development and cataract presence was assessed by visual inspection (see in Methods section, Inheritance assessment). Genomic DNA from the tail biopsy was isolated by phenol-chloroform extraction and ethanol precipitation. Polymerase chain reaction (PCR) was performed using primers for microsatellite markers (initial density 3 markers per chromosome), and PCR products were separated using native polyacrylamide gel electrophoresis (PAGE). Genotypes were called manually. Linkage analysis was performed using MapManager QTX [27].

### *Gja8* sequencing

Tail genomic DNA was isolated as described for linkage mapping. Fragments of *Gja8* were amplified by PCR using the following primers: Cx50_e2a F–TGG AAA GGA AGG TCA CTC CA, Cx50_e2a R–ACA GAG CTC CTC AGC CTC AC, Cx50_e2b F–TCA TCT TCG TCT CCA CTC CA, Cx50_e2b R–GAC ACA AAA GCA ACG GAC AA, Cx50_e2c F–TGT GGT GGA CTG CTT TGT GT, Cx50_e2c R–AGA AGG CAG GGT TTC TTG GT, Cx50_e2d F–ATT TCC CTT TGA CGG AGG TT, Cx50_e2d R–TTG TCA TCG GTT GTC AGC TC, Cx50_e2e F–CCA GAC GGG GAG AAA GTA GA, and Cx50_e2e R–CAG GGC AGG CAT ATG AAA CT. Primers were designed using Primer3 [28]. PCR fragments were analyzed by electrophoresis and sequenced directly using PCR primers and BigDye Terminator v3.1 Cycle Sequencing Kit (Applied Biosystems, Foster City, CA), and the sequencing products were analyzed using ABI PRISM 310 Genetic Analyzer (Applied Biosystems, Foster City, CA). DNA sequences were deposited in GenBank under accession numbers EU445788-EU445793.

### Histological examination of the lens

Whole eyeballs were taken for light microscopic evaluation. Samples were fixed for 12 h in Karnovsky’s solution (2% formaldehyde, 2.5% glutaraldehyde in 0.08 M sodium cacodylate buffer, pH 7.4, with 20 mg CaCl_2_/100 ml) at 4 °C. After repeated washing in 0.1 M sodium cacodylate buffer, a prolonged dehydration in graded series of ethanol, and immersion with acetone and toluene was performed. The samples were then immersed in prepolymerized epoxy resin (Epon 812) according to routine technique and embedded in gelatine capsules. Semi-thin sections (1–2 μm) were prepared on an LKB Pyramitome or Ultrotome III (LKB, Stockholm, Sweden) and stained with toluidine blue.

## Results

### *Dca* inheritance and phenotypes

A cataract phenotype arose spontaneously in our colony of inbred SHR/OlaIpcv rats.

We crossed the founder (with cataract) to SHR. The offspring displayed either a cataract or normal phenotype in 1:1 ratio (four litters, ratio 28:26, χ^2^=0.07, p=0.79). Next, we intercrossed the animals with cataract and obtained animals with a normal phenotype, a cataract phenotype, and a novel phenotype that we describe as microphthalmia. The ratio was 1:2:1, respectively (six litters, ratio 13:32:17, χ^2^=0.58, p=0.75). Crossing animals with microphthalmia to wild type SHR yielded exclusively offspring with cataract (six litters, n=48). Crossing two animals with microphthalmia resulted in homogeneous offspring with microphthalmia (four litters, n=40). The most plausible explanation of these findings is a monogenic Mendelian inheritance in autosomal semi-dominant fashion. We speak about semi-dominant inheritance as there is a pronounced difference between the phenotype of dominant homozygotes and heterozygotes. We named this mutation, *Dca* (for dominant cataract). The SHR-*Dca* strain is coisogenic with SHR/OlaIpcv.

**Figure 2:**
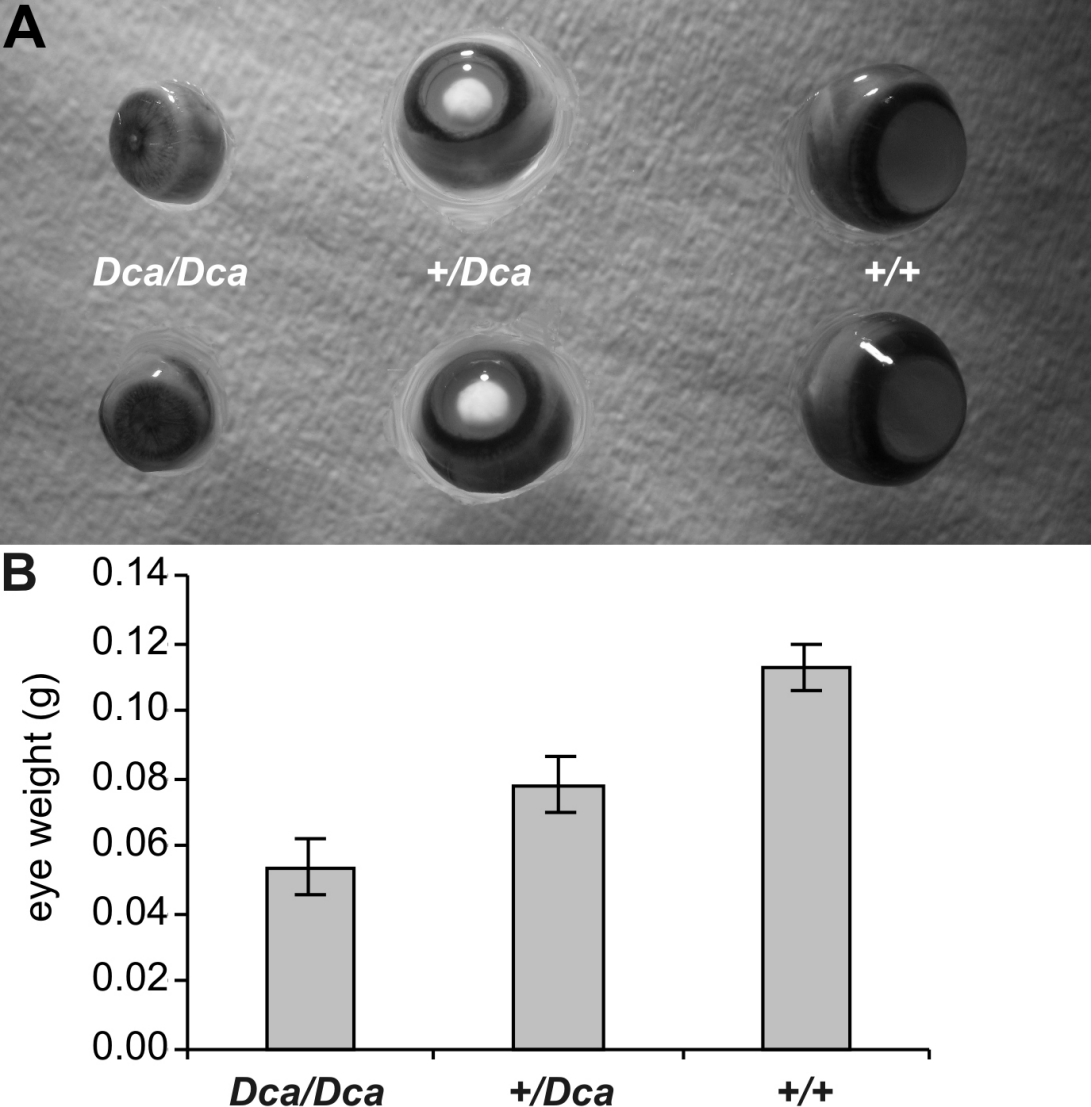
Eyes of *Dca* rats. **A**: Whole eyeball preparations from mutant homozygotes (*Dca/Dca*), heterozygotes (*+/Dca*), and wild type controls (+/+) are shown. Heterozygotes show well developed nuclear cataract while the mutant homozygotes display microphthalmia. **B**: Eye weight is shown for each (mutant homozygotes, heterozygotes, and wild type controls). The data represent means±SEM of the average weight of the left and right eye. The differences are significant (ANOVA F=290.7, p=0, post-hoc comparison with Spjotvoll-Stoline honest significant difference test yielded p=0.00011 among all pairs). The *Dca* allele decreases eye size in an approximately additive manner. *Dca/Dca* homozygotes, n=52, 0.0538±0.0012 g; +/*Dca* homozygotes, n=51, 0.0781±0.0012 g; WT=+/+ (wild-type) controls, n=13, 0.1128±0.0019 g.

Heterozygous animals presented with well developed nuclear cataracts (of the pulverulent type), and they also had smaller eyes. Homozygotes had marked microphthalmia and the lens opacity was not so prominent (Figure 2A). Eyes of the *Dca/Dca* homozygotes weighed about half of the weight of the controls while the heterozygotes had an intermediate reduction in eye size (Figure 2B).

### *Dca* maps to chromosome 2

**Figure 3:**
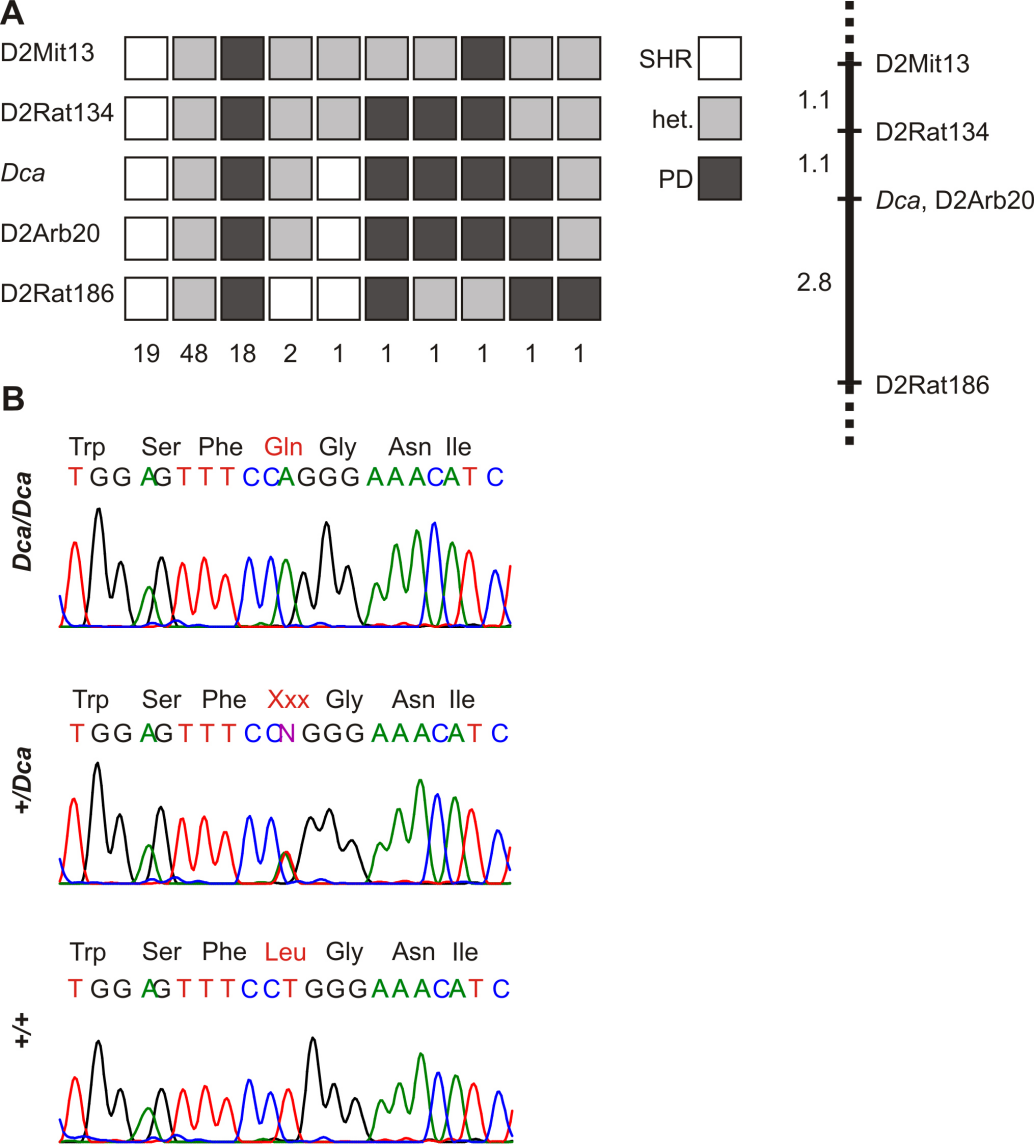
Identification of L7Q mutation in connexin 50 of *Dca* rats. **A**: F2 (SHR-*Dca* x PD) animals (n=93) are categorized according to their genotypes, with number of animals in each category below. Alleles of SHR-*Dca* origin are indicated in white, alleles of PD origin are in dark gray, and heterozygous state is indicated by medium gray color. The corresponding linkage map with distances in cM (Kosambi map function) is shown on the right. The *Dca* gene is inherited in a Mendelian fashion as semi-dominant (ratio in F2 1:2:1, microphthalmia:cataract:normal, χ^2^=0.96, p=0.62). *Dca* maps to chromosome 2. There is no recombination between *Dca* and D2Arb20. The critical interval containing the *Dca* gene is localized between D2Rat134 and D2Rat186 and spans 3.9 cM. The corresponding chromosomal segment has 8.6 Mbp. **B**: Sequencing of the *Gja8* gene is shown. A mutation (T to A transversion in codon 7 of Cx50) results in the substitution of leucine for glutamine (L7Q) in *Dca/Dca* animals. +/*Dca* heterozygotes are also heterozygotes for L7Q.

Using linkage mapping in F2 hybrids (SHR-*Dca/Dca* x PD, n=93), we show the mutation maps to chromosome 2 (Figure 3A). *Dca* is localized to 3.9 cM segment, flanked by markers D2Rat134 and D2Rat186. There is no recombination between *Dca* and D2Arb20. The corresponding chromosomal segment has 8.6 Mbp and contains the *Gja8* gene, which encodes connexin 50 (α8 connexin, Cx50).

### Connexin 50 mutation L7Q

We sequenced the *Gja8* coding sequence and found a T→A transversion in codon 7 in *Dca/Dca* homozygotes. This mutation results in a non-conservative amino acid substitution where leucine at position 7 is replaced by glutamine (L7Q). L7Q cosegregates with 10410 the mutant phenotype. *Dca/Dca* homozygotes (microphthalmia and hypoplastic lens) are homozygotes for L7Q. Heterozygotes +/*Dca* (nuclear cataract, mild microphthalmia) are heterozygotes for L7Q (Figure 3B). The coisogenic strain, SHR, and the three other inbred rat strains (BN, PD, and CHOC) do not have the mutation.

### Impairment of the lens structure in *Dca* rats

**Figure 4:**
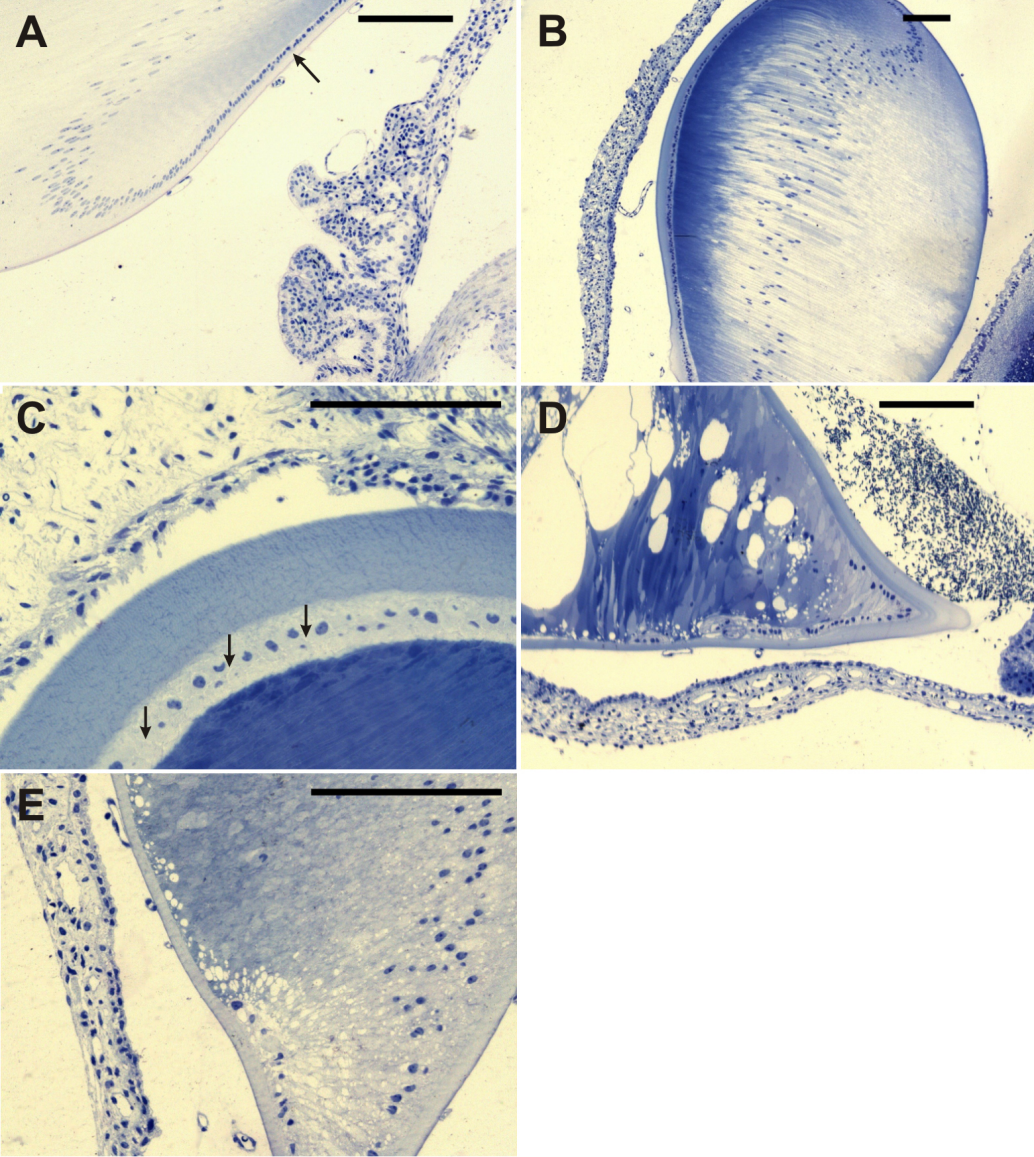
Histological examination of the lens. Epon semi-thin sections were stained with toluidine blue. **A**: The control animal is shown. A single layer of the cuboidal epithelium (arrow) transforms at the equator into elongated cells representing future lens fibers. **B**: The lens of *+/Dca* heterozygotes corresponded in shape nearly completely with that of control animals. At low magnification, the most pronounced feature of the lens is marked heterogeneity in the stainability of its fibers. The lens capsule has often variable thickness and is almost regularly thicker at the anterior face. Persisting vascularization in the posterior eye chamber can be observed. **C**: In *+*/*Dca* heterozygotes, at the higher magnification and tangential section plane orientation, irregularly dilated intercellular spaces between anterior cuboidal epithelial cells (arrows) and newly formed lens fibers can be demonstrated. **D**: Severe alterations of lens development were found in homozygous *Dca/Dca* animals with microphthalmic eyes. The equatorial region forms a regularly sharp angle with a distinct thickening of the lens capsule. The anterior epithelium is discontinuous, and sometimes there are only isolated groups of epithelial cells visible at the anterior face of the lens. Abundant dilatations of intercellular spaces can be seen in some lens regions as great vacuolar formations. Also, stainability of lens fibers increases toward the central part. **E**: Higher magnification of *Dca*/*Dca* lens reveals findings similar to those in **D;** missing continuity of the anterior cuboidal epithelium and striking disturbances in organization and arrangement of cell to cell contacts.

Histological examination of the lens shows relatively normal lens shape and structure in *+*/*Dca* heterozygotes (Figure 4A; *+/+* control animal, 4B and C; *+/Dca* animals with cataract). However, the lens fibers of cataract lenses are markedly different in their stainability, and the lens capsule is irregularly thickened in comparison with control lenses. The intercellular spaces in the anterior epithelium are irregularly dilated (Figure 4C), which is in accord with the expected impairment of gap junction formation between the epithelial cells due to Cx50 mutation. In *Dca/Dca* homozygotes, the lens is markedly smaller (hypoplastic) and its shape is irregular (Figure 4D). The anterior epithelium is absent or is discontinuous, indicating its degeneration. Large vacuoles occur in some parts of the lens as a result of abnormal dilatation of the intercellular spaces between the fibers. In addition, thickening of the lens capsule and the differential stainability of the fibers resemble the changes seen in heterozygotes (Figure 4E).

## Discussion

**Figure 5:**
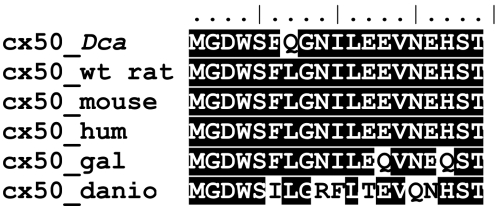
Conservation of NH_2_-terminus of Cx50. Evolutionary conservation of the NH_2_-terminal portion of Cx50 indicates that leucine in position 7 is invariably (100%) conserved across vertebrates (identity/similarity shading using the BLOSUM62 matrix). The sequences are as follows: *Dca* – sequence from the microphthalmic rat (SHR-*Dca/Dca*); wt_rat (*Rattus norvegicus*) – NP_703195.1; mouse (*Mus musculus*) – NP_032149.1; hum (human, *Homo sapiens*) – NP_005258.2; gal (chicken, *Gallus gallus*) – NP_990328.1; and danio (zebrafish, *Danio rerio*) – XP_685033.1.

We identified the L7Q mutation by selective sequencing of the functional positional candidate *Gja8*. The nonrecombinant chromosome segment has 8.6 Mbp and contains at least 44 genes besides *Gja8*. However, none of the other genes is a solid functional candidate for cataract formation. The hypothesis, that L7Q mutation is responsible for cataract formation in SHR-*Dca* rats, is supported by complete cosegregation of L7Q with the mutant phenotypes. Moreover, L7Q is absent from coisogenic SHR/OlaIpcv strain as well as from 3 other unrelated rat inbred strains. The substitution resides in the NH_2_-terminal cytosolic fragment of Cx50 (Figure 1). Leucine in this position is conserved across major vertebrate phyla, indicating a vital role in the protein function (Figure 5). Moreover, leucine at the corresponding position is also conserved in most paralog connexins (with the exception of Cx31.1 coded by the *Gjb5* gene, data not shown). Therefore, we conclude, L7Q is most probably the cause of abnormal eye development in the SHR-*Dca* strain, although the conclusive evidence can be brought around only by additional experiments like transgenic rescue.

*Gja8* involvement in eye and lens development is well established. Recessive cataracts are associated with loss of function as demonstrated in gene targeting experiments [23,24] and also V203fs [25] while (semi) dominant cataracts are caused by different point mutations (Figure 1). The mechanism of the lens affliction stems probably from the subunit composition of connexins (and thus transport properties of gap junctions) both in the lens epithelium and fibers This concept is illustrated by replacement of Cx50 by Cx46 by homologous recombination (knock-in). Single copy Cx46 in place of Cx50 (heterozygous replacement) is able to support development of normal lens size, but results in cataract. On the other hand, When only Cx46 is expressed in place of Cx50 (homozygous replacement), the lens is clear, but its size is subnormal. [29]. The same knock-in can rescue the cataracts caused by dominant form of mutant *Gja8* [9].

Gap junctions are functionally essential for multiple tissues. It is not established yet if Cx50 has any extraocular role. However, it is important to note that the L7Q mutation arises in SHR, a model of hypertension. Considering the role gap junctions play in hypertension [30], it will be interesting to investigate possible links between Cx50 and cardiovascular phenotypes.

L7Q represents a new mutation occurring in the NH_2_-terminal cytosolic portion of Cx50. Based on literature evidence (known null alleles versus point mutations in *Gja8*, see Introduction), we hypothesize that L7Q is not a null allele, but the mutant protein incorporates into gap junctions and actively disrupts their functionality. This mutation in accord with other mutations in Cx50 may be invaluable for detailed study of the molecular mechanisms of the protein function and thus the function of the gap junctions. The NH_2_-terminal cytosolic domain is thought to form a part of the voltage sensor of the connexin channel. In particular, mutations introducing a negative charge in amino acids 2, 5, 8, 9, and 10 of Cx32 resulted in the reversal of gating polarity [26]. Thus, it will be very interesting to investigate the gating properties of the channels formed from the L7Q mutant in Cx50 (L7 in Cx50 corresponds to L6 in Cx32). L7Q may also significantly alter the gating properties but probably in a different manner since L7Q mutation does not include a charge shift.

Only few cataract models are available in the rat. These include an unknown gene mapping to chromosome 15 [31], a mutation in connexin 46 [32], and a mutation in connexin 50 [15]. The Cx50 mutation [15] results in a substitution in the COOH-terminal cytoplasmic domain of Cx50 (R340W, see also Figure 1) and differs from *Dca* by its variable late onset, suggesting that L7Q substitution disrupts the function of Cx50 at a functionally more important amino acid residue.

We have identified the mutation that most likely causes cataract formation in SHR-*Dca* rats. This finding may provide fresh insights into eye development, cataract formation, and connexin function.
